# Comparison of Shielding Material Dispersion Characteristics and Shielding Efficiency for Manufacturing Medical X-ray Shielding Barriers

**DOI:** 10.3390/ma15176075

**Published:** 2022-09-01

**Authors:** Seon-Chil Kim

**Affiliations:** Department of Biomedical Engineering, School of Medicine, Keimyung University, 1095 Dalgubeol-daero, Daegu 42601, Korea; chil@kmu.ac.kr; Tel.: +82-10-4803-7773

**Keywords:** X-ray shielding barrier, eco-friendly, injection molding, pre-mixing, tungsten

## Abstract

During medical diagnoses, X-ray shielding barriers are used to protect against direct and indirect X-rays. Currently, lead is used as the primary material for shielding barriers; however, the demand for eco-friendly shielding barriers has been increasing. Conventionally, shielding barriers are manufactured using a mechanically bonded combination of lead and aluminum; however, in this study, a plastic-based injection-molded product was developed using tungsten as an eco-friendly alternative to lead. A new process technology was required for mixing tungsten—which can be difficult to process—with a polymer. Consequently, the mixing conditions within the injection molding machine and the related compounding technology factors were analyzed. The process technology considered the pre-mixing method using powdery polymer, particle dispersion method, number of screw rotations, and amount of filler input. The product’s shielding performance was then analyzed. The tungsten content of the 2-mm thick barrier manufactured using the proposed method was 90 wt%, and the lead equivalent was 0.321 mmPb. To increase the effectiveness of injection molding in the manufacturing process, specific hourly compounding conditions were proposed. Consequently, the process technology method developed in this study can be considered suitable for manufacturing various shielding barriers.

## 1. Introduction

X-ray shields are safety equipment used in medical institutions during diagnosis and typically include aprons and shielding barriers [1]. When using a mobile X-ray generator in a space that is not equipped with protective facilities—such as a hospital, emergency or operating rooms—an apron and a radiation shielding barrier are required to protect medical personnel and patients [2]. The primary purpose of a radiation shield used by medical staff or patients in a medical institution is to shield the direct ray and to shield the scattered radiation corresponding to the indirect ray. In the case of Apron, it shields the direct rays around the X-ray generator and shields the scattered rays within 1 m of the generator. Radiation shielding barriers are also used for the same purpose. Radiation shielding barriers currently used in medical institutions are made of lead, a lead plate being placed within the product, the outside of which is made of wood [3]. Since lead is a soft metal, it can be easily processed into a desired shape, the thickness of which can be freely adjusted, giving it the advantage of predictable shielding performance [4]. However, when used in the same form as a shielding barrier, a certain strength must be maintained. However, due to lead’s poor strength, it has a distinct disadvantage in that it must be used in combination with other metals, such as tin. Moreover, lead is harmful to the human body as it is a heavy metal, making it difficult to dispose of after use, so medical institutions are reducing their use of it. Consequently, research into and the commercialization of alternative eco-friendly materials are being promoted [5].

In this study, to reduce the use of lead, tungsten was proposed as a substitute for the manufacturing of radiation shielding barriers. Tungsten has a density of 19.25 g/cm^3^, which is higher than that of lead (11.34 g/cm^3^) and because it is an eco-friendly material, its heavy metal risk is low [6,7]. However, it is less cost-effective than lead, making it difficult to replace all radiation-shielding products in the industry with tungsten. Nonetheless, tungsten is an excellent radiation shielding material, depending on the process technology. To achieve shielding performance, strength and processability similar to that of lead, tungsten can be shaped using a mold or injection mold of the desired shape [8,9]. However, to perform injection molding after mixing the polymer and tungsten, a method for manufacturing the pellet containing the shielding material must be developed [10].

Therefore, a technique has to be developed to uniformly disperse inorganic tungsten particles into a resin, which is an organic polymer. The dispersion technology used for shielding materials directly affects the uniformity and reproducibility of their shielding performance [11]. In general, when mass-producing radiation shielding barriers or performing repetitive production processes, a constant ratio of tungsten to polymer is applied [12]. However, when performing this method, the tungsten dispersion area must be controlled so that a similar amount can be equally dispersed, minimizing the agglomeration of the material. In general, since polymers and metal particles do not have good affinity, aggregation occurs even when the particles of the shielding material are well dispersed, making it difficult to maintain a stable dispersion structure within the shield [13].

Rather than using a single process of directly mixing the shielding material and polymer to produce a sheet or plate, this study aims to construct a pellet—that is, a shielding material—through compounding, considering various barrier film shapes [14,15]. The manufactured product can be achieved by evenly dispersing the shielding material within the pellet to maintain the same shielding performance. In this study, tungsten microparticles were used as the shielding material, and polyamide nylon resin (PA66) was selected as the polymer material. When manufactured as a shielding barrier, the product must exhibit good mechanical strength, and PA66 is excellent in this respect [16]. A core tenet of the injection molding process is to set the process conditions to facilitate easy injection molding while maintaining good mechanical and chemical properties and a reliable compounding process for material mixing [17].

In this study, the tungsten particle dispersion method was developed in the material mixing compounding process, and the shielding performance was evaluated. The mixing conditions are important to ensure the even dispersion of tungsten particles in PA66, a plastic material that can be processed into various shapes. In addition, based on the increase or decrease in tungsten content (wt%) during this process, the ease of extrusion and injection may be affected, as well as the mechanical properties [18,19].

In previous studies, most of the shielding material content was limited to 30–50 wt%, and this limitation was due to extrusion and injection processes [20,21]. Therefore, this study attempted to determine the optimal conditions by controlling the mixing time and content of the shielding material to develop mixing technology and improve mechanical properties. The injection stability of the shielding material implies dispersibility. This study was conducted to solve the dispersibility problem by using the conditions of the pretreatment and mixing processes.

Finally, the dispersion of tungsten within the radiation shielding barrier was confirmed by visual analysis using scanning electron microscopy images. Moreover, the shielding performance of the shielding barrier manufactured using the pellet was compared and evaluated based on radiation energy to confirm the effectiveness of the process in terms of shielding performance. The conditions of the high-content pellet manufacturing method and methods to improve the shielding performance of the diagnostic X-ray shielding barrier presented here will enable the manufacture of various types of shields.

## 2. Materials and Methods

During X-ray diagnostics, the radiation generated and thus the shielding area, can be divided into the direct ray and indirect ray area [22]. Apron shielding fabric worn by medical personnel is manufactured for the shielding of direct X-rays and should exhibit a performance of 0.25 mmPb or better based on the minimum lead equivalent [23]. Appropriate process technology is required to increase the thickness or internal density of the shield, as the shielding performance improves only when the material is contained, as far as possible, within the same area inside the shield. In this study, the shielding performance was at least 0.30–0.50 mmPb, with the intention of manufacturing a radiation shielding barrier that could shield both direct and indirect rays in diagnostic X-ray areas.

In general, the density of the radiation shield means the mass per unit volume—that is, if the material density within the shield is high, the interaction probability between the particles of incident radiation and the particles of shielding material increases [24]. Therefore, the incident radiation interacts with the shielding material while passing through the shield, and its intensity is attenuated, as in Equation (1), according to the Lambert–Beer law [25]:

(1) $$I=I_{0}e^{-\left(\frac{\mu }{\rho }\times \rho t\right)},$$

where $I_{0}$ and I denote the unattenuated incident photon and attenuated transmitted photon intensities, respectively, t (cm) denotes the thickness of the shield, μ (cm^−1^) denotes the linear attenuation coefficient; and ρ (g/cm^3^) denotes the density of the shield. Therefore, considering the polymer material and tungsten particles used in this study, the mass decay coefficient ($\frac{\mu }{\rho }$) can be expressed as follows [26]:

(2) $$\frac{\mu }{\rho }=\sum_{i}{𝓌}_{i}\frac{\mu }{\rho },$$

where ${𝓌}_{i}$ denotes the quantity of shielding material within the shield. Therefore, it can be assumed that an increase in the content of tungsten particles would be the most effective method for improving the density within the shield.

Tungsten was used as a shielding material in this study, having a high hardness and a melting point of 3422 °C, making general processing difficult. It can be challenging to maintain the consistency of particle distribution within the injection molding machine, as it is hard to apply the extrusion and injection processes through the screw extruder [27]. However, the more particles there are, the more likely the shielding performance can be improved. Consequently, a technique for compounding the content of tungsten particles in the pellet—which was the raw material of the shielding barrier—from at least 50–90 wt% was required.

The following process was used to overcome the limitations of existing methods so that particles could be well-dispersed while increasing the tungsten content inside the pellet. First, the resin, which is the base material, was ground into a powder. This was done to reduce the non-uniformity between the resin and tungsten particle sizes during the mixing process; the grinding process was hypothesized to enable uniform mixing. Second, a pre-mixing process—that is, the mixing of the powdered polymer and shielding material—was applied for uniform mixing of the two materials and improved dispersibility. Finally, the mechanical conditions within the screw extruder were set. The inside of the injection molding machine refers to the pellet mixing manufacturing process. By analyzing the correlation between screw speed and injection time, this study tracked changes based on the mechanical environment to determine the optimal conditions.

**Figure 1:**
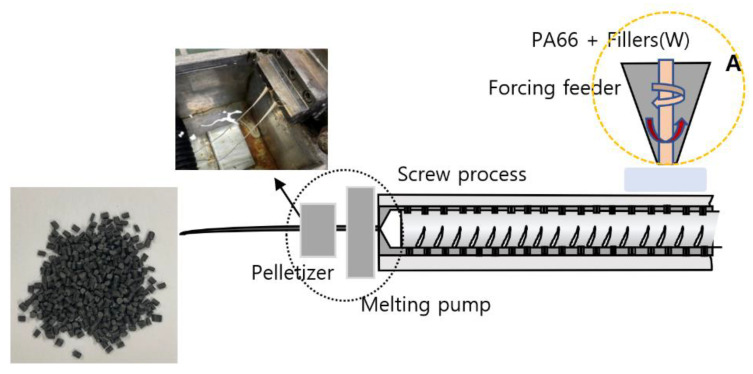
Process of manufacturing pellets: (A) process of mixing tungsten microparticles and powdered PA66 polymers.

The pre-mixing process technology, which mixes the powdered base and the shielding materials, is expected to improve dispersibility when compared to mixing using a liquid base. Figure 1 shows the compounding structure for manufacturing pellets using a single screw extruder. The equipment used for compounding was a Ko-Kneader (2018, HDCK–D10, Incheon, Korea). The Ko-Kneader is a modified single screw extruder that can perform forward, backward and rotational movements simultaneously to uniformly disperse the shielding material during the mixing process.

Through this process, PA66 and tungsten particles were mixed again in the injection machine to increase the dispersibility of the particles. There is sufficient space in the injection molding machine screw to mix the materials, permitting mixing at different time intervals. In general, to improve the distribution of metal particles in polymer materials, the speed and residence time of the screw extruder are important [28]. The dispersion strength (D; mixing intensity) of the shielding material based on the rate (υ; shear rate) and time (t; residence time) of the screw extruder during the mixing process can be expressed as follows [29]:

(3) D=υ·t,

Consequently, if the number of screw rotations within the injection molding machine increases or the average screw running time increases, the degree of dispersion of the shielding material particles also increases. The residence time (*t*) of the material within the screw extruder can be expressed based on the space ($V_{0}$; free volume, V; filling volume) and filling level (f; filling level) in the screw extruder and can be expressed as follows [30]:

(4) $$t=\frac{f\cdot V_{0}}{V},$$

Consequently, if the space within the screw extruder remains constant, the filling volume increases. The filling volume itself is affected by the filler input volume and the rotational speed of the screw extruder, and it could be expected that the slower the screw speed and the greater the input volume, the more difficult the dispersion. A previous study suggested speed control based on the front and rear directions of the screw, but in this study, a method to increase dispersion by controlling the flow rate per hour of the polymer resin and the input speed of the mixture was proposed [31]. Therefore, in this study, the degree of dispersion based on the input volume of mixed material per hour and the speed of the screw extruder were examined. Dispersibility was evaluated by determining the shielding performance of shields of the same thickness manufactured with up to 100% tungsten.

**Figure 2:**
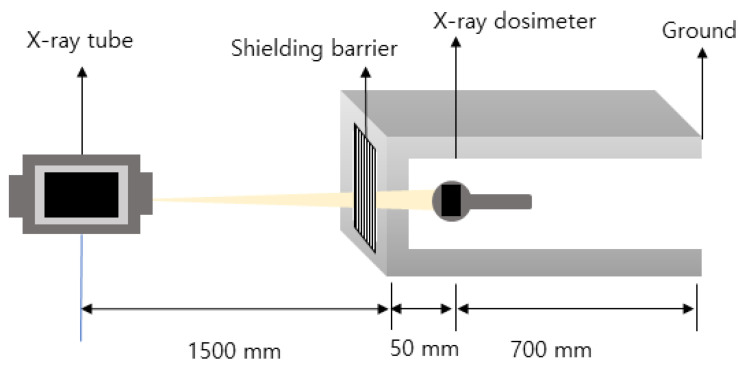
Shielding performance evaluation diagram of X-ray barriers.

In this study, a pellet was manufactured by injection molding (under three process conditions), and a radiation barrier (0.5 m × 0.5 m × 2 mm) was manufactured by extrusion molding. In addition, the dispersibility of the finally fabricated shielding barrier was visually observed using an optical microscope (FESEM; Field emissions scanning electron microscope, Hitachi, S-4800, Tokyo, Japan). The shielding performance evaluation was conducted using the structure shown in Figure 2 [32]. After setting 60, 80, 100, 120 kVp, and 20 mAs using an X-ray generator (Toshiba, E7239, 150 kV-500 mA, 1999, Tokyo, Japan), the average value obtained from 10 experiments was calculated. Since the energy of the X-ray used is not a single energy, the effective energy is used, which can be determined by a half value layer (HVL) measurement method. Therefore, the effective energy was determined by checking the linear attenuation coefficient (μ) and Hubbell’s mass attenuation coefficient ($\frac{\mu }{\rho }$) [33]. The effective energy of X-rays determined by HVL was about 34–60 keV for a tube voltage of 60–120 kVp. The dose detector used (after calibration) was DosiMax Plus 1 (2019; IBA Dosimetry, Schwarzenbruck, Germany).

## 3. Results

**Figure 3:**
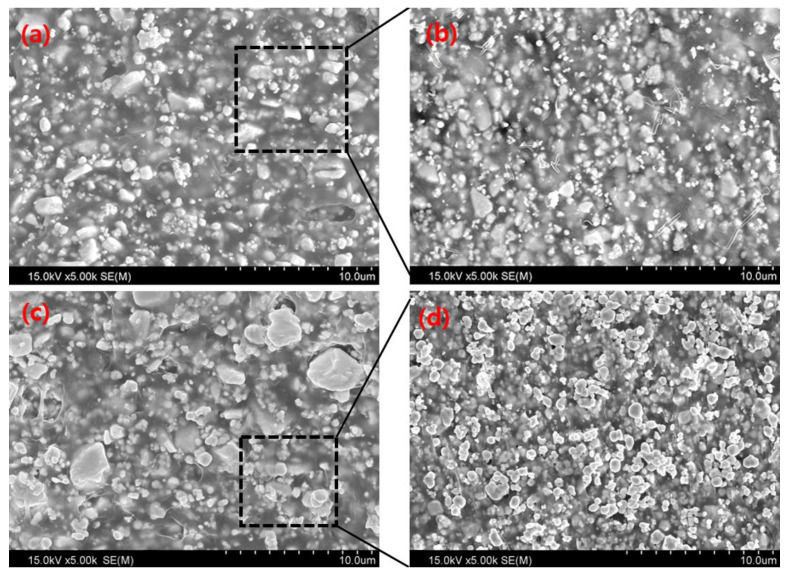
Particle distribution based on the mixing method of tungsten particles and PA66. (**a**,**b**) show the distribution diagrams of particles using a liquid polymer material without using a pre-mixing process, (**c**,**d**) show the particles obtained by pre-mixing a polymer in a powdered state using a pre-mixing process.

The aim of this study was to quantitatively evaluate three technologies to improve the manufacturing conditions and shielding performance of diagnostic X-ray barriers used in medical institutions. First, the dispersion of tungsten particles was verified by evaluating the mixing time of a product manufactured using a pre-mixing process before the mixing and heat treatment of liquid PA66 and tungsten. Figure 3 shows the internal cross-sectional view of a shielding barrier manufactured using the existing process and a shielding barrier manufactured using the pre-mixing process of this study. Figure 3a,b show cross-sectional views of a shielding barrier manufactured by mixing a polymer material (in a liquid state) and tungsten, and Figure 3c,d show cross-sectional views of a shielding barrier manufactured using the proposed pre-mixing process of mixing a polymer material in a powdered state and tungsten. In Figure 3a,b, it can be seen that the distribution of tungsten particles is not uniform, with aggregation of the polymer and tungsten occurring. In Figure 3c,d, the tungsten particles dispersed in the resin are uniformly distributed.

**Figure 4:**
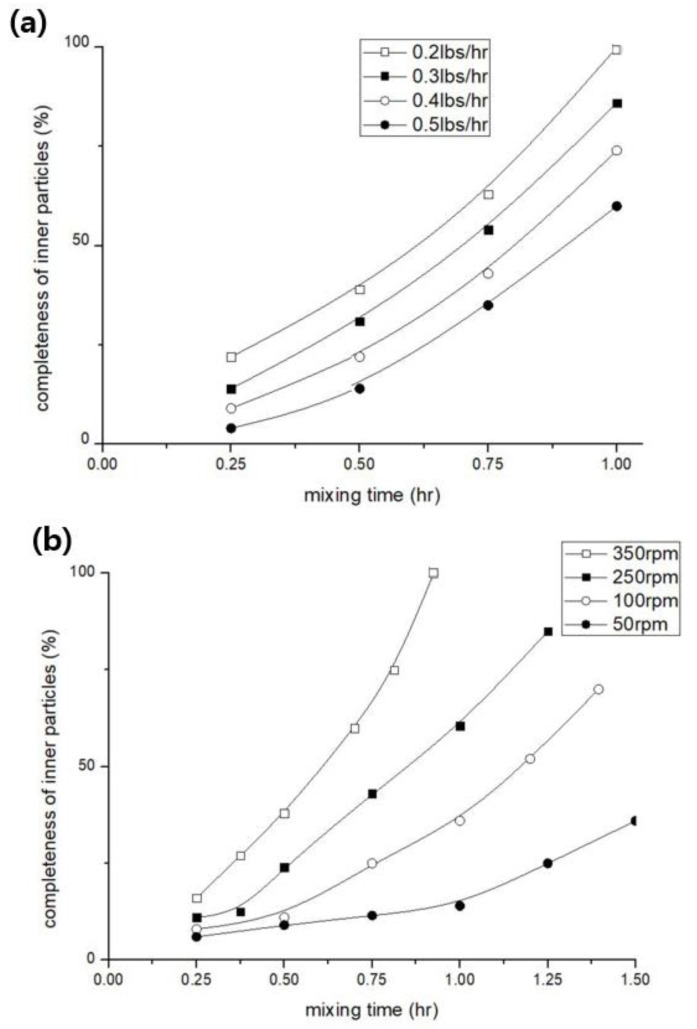
Comparison of tungsten input volume and mixing time based on injection molding machine conditions. (**a**) Mixing time based on the volume of tungsten per hour, (**b**) tungsten mixing time based on the screw rotational speed.

Figure 4 shows a graph of the tungsten particle dispersion based on the mixing conditions within the injection molding machine. Figure 4a shows the filler input volume based on the final input volume of tungsten, and Figure 4b shows the dispersion degree based on the screw rotational speed. The results shown in Figure 4 are based on the pellet produced in this study, the dispersibility being evaluated by visual analysis of the final injection molded product. As can be seen, when the quantity of tungsten per hour is slowly injected and the rotational speed of the screw per hour is increased, a larger quantity of tungsten is injected.

**Figure 5:**
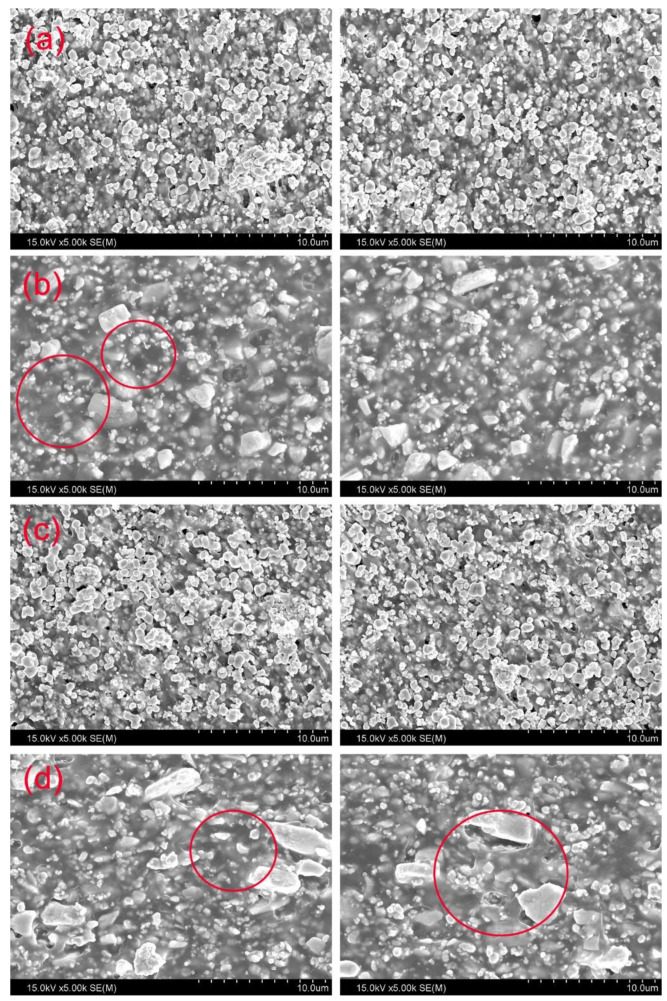
A visual evaluation of tungsten particle dispersion based on the conditions of the injection molding machine. (**a**) Particle distribution of the shield produced using a pre-mixing process by adding approximately 100 g of tungsten per hour, (**b**) Particle distribution of the shield produced by injecting 50% of the total volume of tungsten at approximately 230 g per hour (Red circles indicate voids caused by non-uniform particle distribution), (**c**) Particle distribution of the shield with improved mixing strength by increasing the screw rotational speed (350 rpm) and reducing the mixing time, (**d**) Particle distribution of the shield with reduced mixing strength by lowering the screw speed (50 rpm) and increasing the mixing time (Red circles indicate voids due to polymer voids).

Figure 5 shows the results of evaluating dispersibility based on mixing time and screw rotational speed using a cross-sectional view of the shield. Figure 5a shows the results achieved by controlling the amount of tungsten per hour to 100 g, and Figure 5b shows the results achieved by adding approximately 230 g of tungsten per hour. The tungsten particles are more uniformly distributed in the cross-sectional view of Figure 5a compared to Figure 5b. In addition, Figure 5c shows the particle distribution in the shield with the screw rotational speed increasing to 350 rpm and the mixing time shortening. Figure 5d shows the particle distribution within the shield with a screw rotation speed of 50 rpm and an increased mixing time.

The injection volume of tungsten and the rotational speed of the screw affect the particle residence time distribution and negatively affect the strength of the injection-molded product [34]. As shown in Figure 5c, if the maximum volume of tungsten is injected and the screw speed is increased to improve the affinity of the polymer, the mixing strength can be greatly improved. However, as shown in Figure 5d, if the rotational speed of the screw is lowered and the mixing time increases too much, the viscosity decreases due to polymer decomposition [35]. In this study, the volume of tungsten input was fixed at a maximum of 80 wt% and 90 wt%.

**Figure 6:**
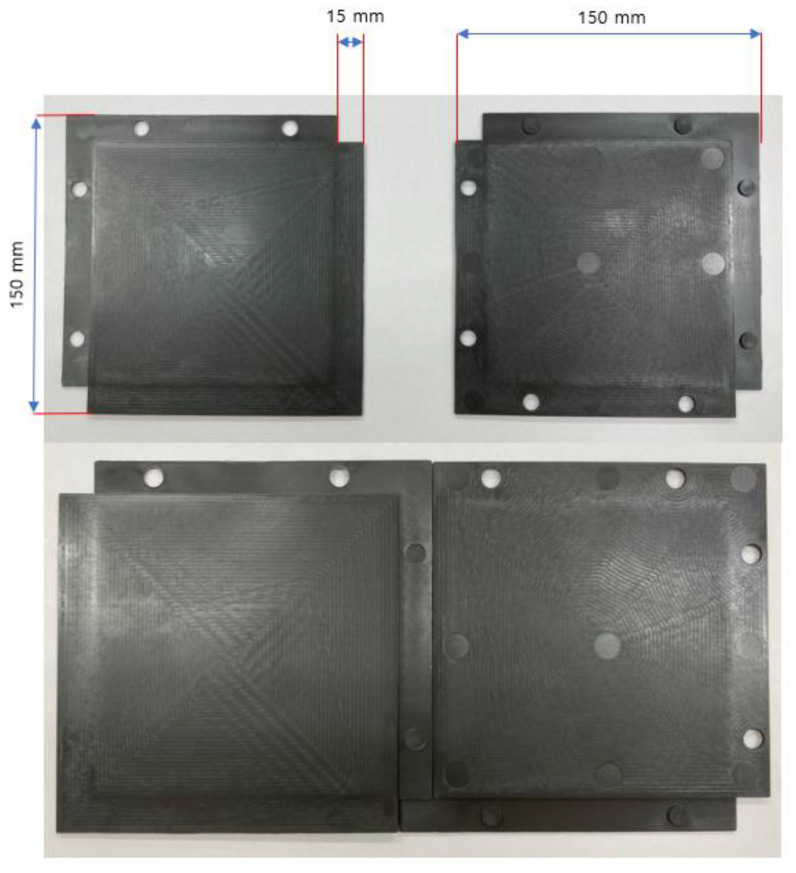
Final shielding barrier panel manufactured by injection molding.

A panel of dimensions 0.5 m × 0.5 m × 2 mm (102 ± 0.01 g) that could be used as a material for a radiation shielding barrier was finally manufactured, as shown in Figure 6. The seam of the panel was manufactured to a thickness of 1 mm so that when two panels were connected, a 2-mm thickness could be maintained, making safe shielding possible.

The X-ray shielding performance results of the manufactured radiation shielding barrier were presented based on the lead equivalent and shielding rate. X-rays of 60–120 kVp for diagnoses typically used in medical institutions were used, and the shielding performance was presented using the lead equivalent compared to the standard lead. Table 1 shows the shielding performance results of standard lead—that is, lead with a purity of 99.9% or more. At 100 kVp, 0.2 mm of lead had a shielding rate of 80.43%.

Table 2 shows a comparison of the shielding performance of the shielding barrier produced by pre-mixing the polymer and tungsten in a powdered state with the shielding barrier produced by mixing the polymer and tungsten in the liquid state. At 100 kVp, there is a lead equivalent difference of 0.084 mmPb in the two products and a shielding rate difference of 4.32%, indicating that the pre-mixing process of powdered polymer and tungsten plays an important role in the pretreatment process, confirming the improved dispersion of tungsten metal particles and shielding performance.

Table 3 shows the results of the changes in content based on the conditions within the injection molding machine. As predicted, the higher the tungsten content, the better the shielding performance. When comparing the 90 wt% product with the maximum tungsten content increased with the technology developed in this study and the existing product with the content limited to 50 wt%, a lead equivalent difference of 0.201 mmPb was shown at 100 kVp. Consequently, it is desirable to increase the content of the shielding material if there is no change in the intrinsic properties of the polymer material.

Table 4 compares the shielding performance based on the mixing time within the injection molding machine. The mixing process for 30 min and the mixing process for 1 h are compared, and the change in particle dispersibility and the affinity of the two materials are compared when the polymer material is mixed for a long time. It can be seen that the degree of dispersion of tungsten particles improves and the shielding performance improves as the mixing time increases.

Consequently, the shielding performance of the 2-mm thick tungsten barrier product is 0.321 mmPb (lead equivalent) at 100 kVp, which is more effective than the existing 0.25 mmPb lead shield in terms of thickness and shielding performance.

## 4. Discussion

In practice, the size of shielding barriers is not fixed and depends on their proposed use. However, the usable distance of shielding barriers is usually less than 1 m, so they are used to protect users when taking close-up shots [36]. Although aluminum, tin and copper can be used instead of lead, it is most effective to use a material with excellent workability to realize the required shape [37]. In this study, a shielding barrier was manufactured using tungsten-based plastic injection molding as the processing method.

Powder injection molding (PIM) is a common injection molding technique, but when tungsten is selected as a shielding material, the processability is low and accessibility is somewhat inadequate [38]. In the case of manufacturing existing shielding sheets, a method of mixing urethane and rubber with the shielding material using thermal processing technology is used [39], a method better understood to be a calendaring sheet manufacturing process rather than a powdered injection molding process. The PIM process makes it easy to manufacture small shields with complex shapes that can be difficult to manufacture by pressing or machining [40].

The method proposed in this study is a combination of plastic injection molding and powder metallurgy, which maximizes the content of shielding materials based on the desired shape and size. While it can be difficult to utilize more than 80 wt% of tungsten in existing shielding sheets, it is crucial to increase the content to meet the strength rather than flexibility requirements in the production of the shielding barrier [41].

Consequently, to improve the shielding performance of the radiation shielding barrier, it is important to increase the content of the shielding material—that is, the shielding metal particles should be appropriately dispersed and positioned in the polymer material. If the amount of shielding material is reduced, even for the same mixing time, the mixing ratio of the resin increases, resulting in an imbalance in the resin content [42]. However, this study confirmed that some loss and deformation of the resin occurred due to heat treatment problems as the working time increased. The quantity of affected polymer material was very small and did not affect the overall mass and density but could affect the dispersion of tungsten. However, it did not affect the bonding strength of the polymer material but rather helped the dispersion of the tungsten particles, resulting in improved shielding performance.

Problems can be associated with the aggregation of the polymer material during the manufacture of the polymer radiation barrier. Consequently, in this study, the screw rotational speed was set to 300–350 rpm to prevent agglomeration and to increase the bonding force of the metal shielding material. In addition, to reduce the aggregation phenomenon of the PA66 matrix resin, the tungsten input volume was set from 50 wt% to a maximum of 90 wt%, and the rate per hour was gradually increased. However, if a high content of filler (which is a raw material used in the injection molding process) is added, it can be difficult to obtain a sufficiently high tungsten content (of more than 40%) due to the uneven mixing as a result of the differences in particle size, making pulverizing the base resin a very important part of the process. Consequently, this study demonstrated a method for this, emphasizing the importance of the pre-mixing process in the mixing of metal materials.

In general, the faster the screw speed, the better the mixing and mechanical properties, although problems such as reduced viscosity and discoloration can occur due to the decomposition of the resin, potentially causing deterioration of the mechanical properties [43]. This processing problem can be solved to some extent by setting an appropriate combination of screws. In other words, to maintain a balance of the resin ratio during the mixing process, the mixing effect can be maintained by processing at a low rotational speed so that the amount of energy transmitted by screw rotation can be used for mixing the two materials rather than raising the temperature of the resin.

The degree of dispersion of the shielding material was visually confirmed using the method presented in this study, confirming that the shielding performance of the final shield manufactured compared favorably with the existing process. The improved results confirmed that the higher the content of the shielding material and the longer the mixing time, the better the dispersion of the particles. A higher screw rotational speed, if not too high, also helps disperse the metal shielding material.

However, in this study, the thickness of the mold was fixed at 2 mm, so there was a limit to the evaluation of the dispersion of the shielding material based on any change in thickness. Mixing time and tungsten content must be considered important issues in the injection molding process to improve the affinity and dispersibility of tungsten particles and polymers. Therefore, to develop an eco-friendly and economical shield in the future, these technologies must be considered.

## 5. Conclusions

A plastic-based X-ray shielding barrier was manufactured via injection molding by mixing tungsten particles (a metal shielding material) with PA66 (a polymer material). The shielding barrier of 2-mm thickness showed a shielding performance of 0.321 mmPb (lead equivalent), achieved by obtaining a high tungsten content of 90 wt% using the pre-mixing of the powdered polymer with the shielding material and optimizing the mixing speed and time. Consequently, this study confirmed that injection molding could be a suitable diagnostic X-ray eco-friendly shielding barrier manufacturing process for improving the shielding performance of X-ray barrier shields.

## Figures and Tables

**Table 1:** Shielding performance evaluation of standard lead (0.1 mm, 0.2 mm, 0.3 mm).

mmPb	Effective Energy Transmission Dose	34.8 keV (60 kVp)		49.1 keV (80 kVp)		55.3 keV (100 kVp)		60.8 keV (120 kVp)	
		Non	Lead	Non	Lead	Non	Lead	Non	Lead
0.1	Dose (μC/kg)	0.107147	0.0176446	0.2259125	0.0614994	0.3866568	0.13932	0.4853831	0.1873518
	Shielding rate (%)	-	83.53	-	72.78	-	63.97	-	61.40
0.2	Dose (μC/kg)	0.107147	0.0052812	0.2259125	0.0275982	0.3866568	0.0756636	0.4853831	0.1057206
	Shielding rate (%)	-	95.07	-	87.78	-	80.43	-	78.22
0.3	Dose (μC/kg)	0.107147	0.0020149	0.2259125	0.014435	0.3866568	0.0466644	0.4853831	0.065944
	Shielding rate (%)	-	98.12	-	93.61	-	87.93	-	86.41

Evaluated as lead with a purity of 99.9% or higher. Non denotes the dose when there is no shield.

**Table 2:** Comparison of shielding barrier shielding performance with or without the pre-mixing process.

Pre-Mixing	Effective Energy Transmission Dose	34.8 keV (60 kVp)		49.1 keV (80 kVp)		55.3 keV (100 kVp)		60.8 keV (120 kVp)	
		Non	SB	Non	SB	Non	SB	Non	SB
Nothing	Dose (μC/kg)	0.107147	0.0095305	0.2259125	0.0381504	0.3866568	0.080643	0.4853831	0.1086102
	Shielding rate (%)	-	91.10	-	83.11	-	79.14	-	77.62
	Lead equivalent	-	0.109	-	0.114	-	0.124	-	0.126
Processing	Dose (μC/kg)	0.107147	0.004540	0.2259125	0.021955	0.3866568	0.0639504	0.4853831	0.0931302
	Shielding rate (%)	-	95.76	-	90.28	-	83.46	-	80.81
	Lead equivalent	-	0.201	-	0.206	-	0.208	-	0.207

Nothing denotes a barrier that does not use the pre-mixing process, Processing denotes a barrier that uses the pre-mixing process. Non denotes the dose when there is no shield, SB denotes the shielding barrier.

**Table 3:** Comparison of tungsten content change and shielding performance based on the internal conditions of the injection molding machine.

wt%	Effective Energy Transmission Dose	34.8 keV (60 kVp)		49.1 keV (80 kVp)		55.3 keV (100 kVp)		60.8 keV (120 kVp)	
		Non	SB	Non	SB	Non	SB	Non	SB
50	Dose (μC/kg)	0.107147	0.0098710	0.2259125	0.040454	0.3866568	0.0914687	0.4853831	0.1248642
	Shielding rate (%)	-	90.79	-	82.09	-	76.34	-	74.28
	Lead equivalent	-	0.109	-	0.113	-	0.120	-	0.121
70	Dose (μC/kg)	0.107147	0.0064809	0.2259125	0.0227788	0.3866568	0.048207	0.4853831	0.0645309
	Shielding rate (%)	-	93.95	-	89.92	-	87.53	-	86.70
	Lead equivalent	-	0.198	-	0.205	-	0.218	-	0.222
90	Dose (μC/kg)	0.107147	0.0029102	0.2259125	0.008372	0.3866568	0.0228484	0.4853831	0.0259212
	Shielding rate (%)	-	97.28	-	96.29	-	94.09	-	94.66
	Lead equivalent	-	0.298	-	0.309	-	0.321	-	0.329

wt% denotes the tungsten content ratio, the higher the number, the higher the content compared to the polymer material. Non denotes the dose when there is no shield, SB denotes the shielding barrier.

**Table 4:** Comparison of shielding performance based on the mixing time of the same content.

Time (m)	Effective Energy Transmission Dose	34.8 keV (60 kVp)		49.1 keV (80 kVp)		55.3 keV (100 kVp)		60.8 keV (120 kVp)	
		Non	SB	Non	SB	Non	SB	Non	SB
0.5	Dose (μC/kg)	0.107147	0.009355	0.2259125	0.0378537	0.3866568	0.0836229	0.4853831	0.111907
	Shielding rate (%)	-	91.27	-	83.24	-	78.37	-	76.94
	Lead equivalent	-	0.109	-	0.114	-	0.123	-	0.125
1.0	Dose (μC/kg)	0.107147	0.0091022	0.2259125	0.027206	0.3866568	0.0542109	0.4853831	0.0879135
	Shielding rate (%)	-	91.50	-	87.96	-	85.98	-	81.89
	Lead equivalent	-	0.110	-	0.121	-	0.134	-	0.133

The screw speed is 300 rpm, and the tungsten content is the same at 85 wt%. Non denotes the dose when there is no shield, and SB denotes the shielding barrier.

## Data Availability

Not applicable.
